# Test-retest reliability and minimal detectable change for measures of wearable gait analysis system (G-Walk) in children with cerebral palsy

**DOI:** 10.55730/1300-0144.5358

**Published:** 2022-01-13

**Authors:** Melek VOLKAN-YAZICI, Gamze ÇOBANOĞLU, Gökhan YAZICI

**Affiliations:** 1 Department of Physical Therapy and Rehabilitation, Faculty of Health Sciences, Yüksek İhtisas University, Ankara, Turkey; 2 Department of Physical Therapy and Rehabilitation, Faculty of Health Sciences, Gazi University, Ankara, Turkey

**Keywords:** Cerebral palsy, gait analysis, reliability, wearable sensor device

## Abstract

**Background/aim:**

Cerebral Palsy (CP) is the most frequent cause of physical disability in childhood. CP causes primary deficits such as impairments in muscle tone, muscle weakness, problems in selective motor control and secondary deficits such as contractures and deformities. These deficits lead to motor disorders during movement causing limitations in gait. Sixty percent of children with CP can walk independently despite these problems, however, they present with various gait abnormalities. Gait analysis is used in the quantitative assessment of gait disturbances providing functional diagnosis, assessment for treatment, planning, and monitoring of progress. G-Walk is a wearable sensor device which provides quantitative gait analysis via spatiotemporal parameters and pelvic girdle angles. In literature, there is no study investigating the reliability of the G-Walk in children with CP. The purpose of this study was to confirm the test-retest reliability of a commercially available body-worn sensor ‘BTS G-WALK sensor system’ for spatiotemporal gait parameters in children with CP.

**Materials and methods:**

Fifty-four children with CP (mean age: 9.19 ± 3.49 years), Gross Motor Function Classification System (GMFCS) level I–II completed the test-retest protocol with 5 days between tests. The test-retest reliability was calculated using intra-class correlation coefficients (ICC). Minimal detectable changes were calculated using standard error measurements.

**Results:**

According to the analysis, ICC varied from 0.799 to 0.977 in all of the gait parameters. The statistical analysis showed that all G-Walk parameters’ measurements were found to have almost perfect test-retest reliability.

**Conclusion:**

The G-Walk was found to be reliable in gait parameters for children with CP between ages 5 and 15, in GMFCS level I–II. A gait analysis carried out with the G-Walk system is a reliable method to assess gait in children with CP in a clinical setting.

## 1. Introduction

Cerebral palsy (CP) is caused by a nonprogressive injury in the developing brain, which leads to problems in functional mobility, posture, neuro-musculoskeletal functions, and gait [1,2]. Around 75% of children with CP are ambulatory and 60% of children with CP are able to walk independently however, they have gait problems such as excessive knee flexion, stiff knee, crouch gait, or equinus which affects the quality of gait [3,4].

Normal gait function is one of the most complicated dynamic tasks of the musculoskeletal and neurological systems. In cerebral palsy, primary disorders of these systems may lead to secondary disturbances in gait. Gait analysis is used in the quantitative assessment of gait disturbances providing functional diagnosis, assessment for treatment, planning, and monitoring of progress [5]. In gait analysis, a large amount of quantitative data concerning the gait characteristics of a patient is analyzed. The assessment of these data can be performed via standardized clinical videos, recorded with numerical video cameras used in conjunction with optical 3D systems [6]. These assessment methods are frequently used, however, they have their disadvantages; observational/video gait analysis was found to be inaccurate [7–9], 3D gait analysis systems require more time, technical expertise and equipment than is available in the average physiotherapy department and are costly [10]. Due to these drawbacks, wireless inertial sensors, which are wearable sensor devices (WSD) are now used in gait assessment. WSDs are electronic devices that are worn on the surface of the skin, where they detect, analyze, and transmit information concerning body signals. WSDs are easy to use, lightweight and cost-effective. Since these devices are wireless, unrestricted movement is enabled [11].

**Figure 1:**
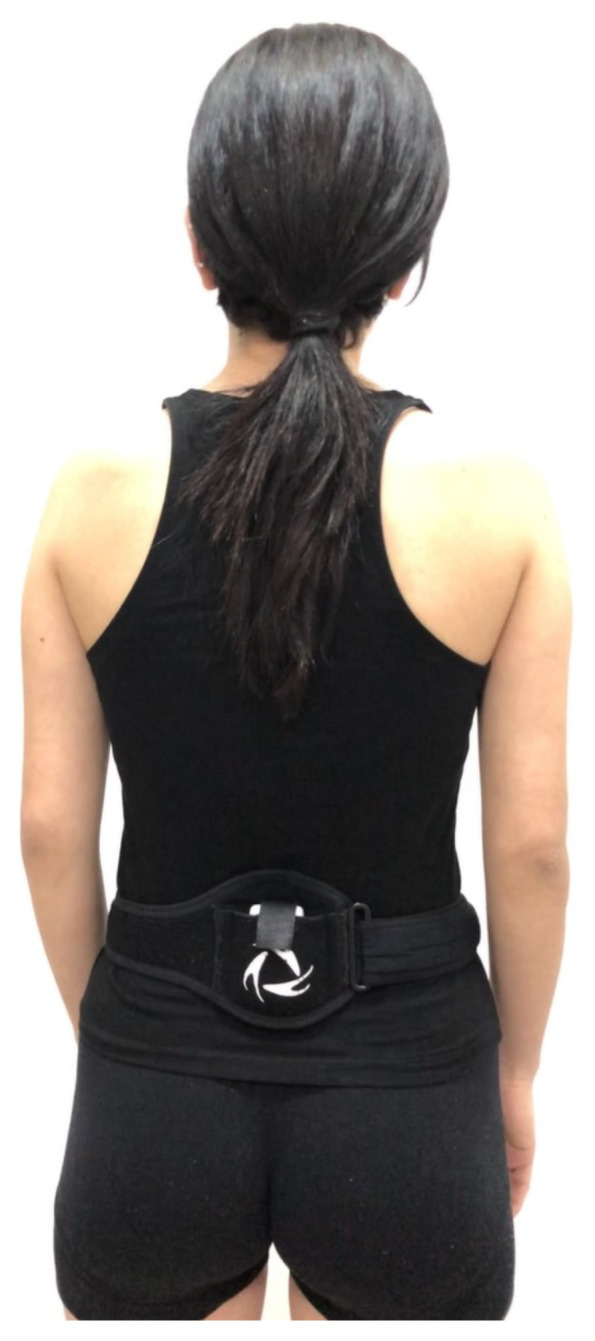
The G-Walk is placed on an elastic belt and worn on the waist.

The BTS G-WALK sensor system (G-Walk) determines spatiotemporal parameters as well as all pelvic movement (rotation, tilt and obliquity) during gait. The G-Walk, is a WSD which is placed on an elastic belt and worn on the waist of the person being evaluated. The device is placed on the waist with the center of the device at the fifth lumbar vertebrae and the patient is completely free to walk, run and jump [11]. The G-Walk can be seen in Figure 1. The system worn by the subject provides a series of parameters that analyze various movements including walking, running and jumping. The acquired data is transmitted to a computer through a Bluetooth connection. For the analysis, all measurements are calculated based on the person’s height and movements. Therefore, it is necessary to enter the height of the person prior to assessment. The height of the subjects is used by the calculation algorithm to properly identify the gait parameters. The software used is BTS G-Studio. G-Studio is a simple and easy-to-use software that can manage different acquisitions and automatically elaborate and report different analysis protocols [12]. At the end of each analysis a report containing all the parameters is created automatically by the software.

The G-Walk was recently introduced as a multipurpose testing and treatment device for the assessment of gait. Before using such devices for clinical interpretation, the reliability must be investigated. In literature there is one study evaluating the reliability of the G-Walk. In this study by De Ridder et al., the concurrent validity of the G-Walk on gait parameters in healthy subjects was assessed. They have concluded that, the G-Walk is reliable for all measured spatiotemporal parameters and has excellent concurrent validity for speed, cadence, stride length, and stride duration [11]. However, this study only focuses on healthy subjects making it impossible to generalize the results for specific populations. It is unknown whether the G-Walk is a reliable clinical assessment tool for children with CP. The measurements should be reproducible, stable, accurate, capable of distinguishing between normal and abnormal conditions. As a portable low-cost device, G-Walk may be beneficial in the assessment of gait in CP, may assist in observational gait analysis and investigating the gait pattern progression in the clinic. Therefore, the purpose of this study was to confirm the test-retest reliability of a commercially available body-worn sensor ‘BTS G-WALK sensor system’ for spatiotemporal gait parameters in children with CP.

## 2. Materials and methods

### 2.1. Participants

Fifty-four children (25 females, 29 males) completed the test-retest protocol with 5 days between tests. Children between 5–15 years of age who were in level I (able to walk in all settings with some balance and coordination impairments) or level II (walking is limited in some settings) according to the Gross Motor Function Classification System (GMFCS), had spastic CP on one (unilateral CP) or two sides (bilateral CP) of the body, could walk unassisted and could cooperate were included in the study. Children in GMFCS level I and II were included in this study because children in these levels were able to walk without support. The demographics and functional characteristics of the children can be seen in Table 1. Those who had received botulinum toxin injections in the past 6 months or those who had undergone an orthopedic surgery involving the lower extremities were excluded from the study. Before recruiting children for our study, a power analysis was performed. The sample size was calculated as 53 patients by using G-power version 3.1.9.5. according to the study by De Ridder et al. (effect size 0.17, α error probability = 0.05, and 80% power) [11]. Informed consent forms were obtained from the patients and their caregivers stating that they were willing to participate in the study. This study was approved by the ethical committee of X University and the authors conformed to the ethical guidelines of the 1975 Declaration of Helsinki.

### 2.2. Study design and procedures

This study was a cross-sectional design which included patients with cerebral palsy who could walk independently. Upon arrival at the first test session, the caregivers of the participants filled out informed consent and medical history form that included demographic information and answered questions determining inclusion/exclusion criteria for the study.

The gait parameters of the participants were evaluated using the G-Walk sensor system (BTS G-Walk BTS Bioengineering Company, Italy). G-Walk is built with a triaxial accelerometer 16 bit/axes with multiple sensitivity, a triaxial magnetometer 13 bit (±1200 μT) and a triaxial gyroscope 16 bit/axes with multiple sensitivity (±250, ±500, ±1000, ±2000°/s). All data were collected using a sampling frequency of 100 Hz. The device is placed on an elastic belt and worn on the waist of the person being evaluated, with the center of the device at the fifth lumbar vertebrae [11]. To ensure the correct placement of the device, the L4–L5 intervertebral space was palpated via the posterior superior iliac spines. After the G-Walk was placed, the children were asked to walk calmly at normal speed, on a 7-meter track and to return to the starting position. The boundaries of the track were determined with colored lines. The examiners walked alongside the children when necessary to ensure safety and maintain walking velocity. A successful trial was characterized by the participant completing the 7-meter track and returning to the starting point. Any extra or unexpected movements such as sneezing, coughing, stumbling or alterations in the velocity (such as running or almost stopping) of the gait deemed the trial unsuccessful, and thus was repeated.

The G-Walk provided quantitative analysis for the performance of walking via spatiotemporal parameters as well as pelvic movements during gait. It also enables analysis of the pelvis angles, providing a functional analysis of disorders in gait caused by neuromuscular diseases [13,14]. The tilt of the pelvis in the sagittal plane in the flexion-extension direction, the obliquity of the pelvis in the coronal plane, the angles of rotation of movement in the transverse plane and the symmetry values of the right and left sides were obtained. While the symmetry index ranges from 0 to 100, a value closer to 100 indicates that the gait is more symmetrical [15].

The children were tested after a practice trial, until we obtained two successful trials per test session. The tests were performed by the same examiner within an interval of five days (test-retest). The second successful trial of each session was included in the analysis.

### 2.3. Data analysis

Statistical analysis was conducted using the SPSS 22 computer software system. The variables were investigated using analytical methods (Kolmogorov-Smirnov test) to determine whether or not they are normally distributed. Descriptive analyses were presented using means and standard deviations (SD) for normally distributed variables. Systematic differences were identified using a paired T-test. Statistical significance for this study is based on the p < 0.05 level. For the reliability, test-retest analysis intra-class correlation coefficient (ICC) with absolute agreement and 95% confidence interval (CI) were determined between the first and second assessment. The minimal detectable change (MDC), also referred to as the “smallest detectable difference,” is an absolute measure of reliability, which accounts for various sources of variability in defining a confidence interval in units of the measure. MDC is the smallest change you can measure above this systematic error. It is important to calculate MDC because the MDC is the minimum amount of change in a subject’s score that ensures the change is not the result of measurement error. MDC was calculated by multiplying the SD of the difference with 1.96. When evaluating interventions, the pre-post difference must be larger than the MDC to express real improvement [16]. The standard error of measurement (SEM) also provides a measure of variability but was primarily used for calculating the MDC. SEM values were calculated as follows: SEM = SD × √(1 – ICC), with SD representing the standard deviation of the measure [16]. The ICC values were defined as; higher than 0.81 was almost perfect, 0.61–0.80 was high, 0.41–0.60 was moderate, 0.21–0.40 was fair [17].

## 3. Results

Data analysis was performed with the data obtained from 54 participants and the demographic statistics are shown in Table 1. Mean and SD values of test and retest G-Walk measurements of affected and less affected sides are presented in Table 2. Table 3 demonstrates ICC, 95% CI, SEM, and MDC. According to the analysis, ICC varied from 0.799 to 0.977 in all of the gait parameters. The statistical analysis showed that all G-Walk parameters’ measurements had almost perfect test-retest reliability. There was no significant difference between test and retest mean scores according to paired T-test for any G-Walk measures, which indicates absence of any systematic bias (p > 0.05).

## 4. Discussion

This study provided evidence related to test-retest reliability and MDC values of the G-Walk sensor system for spatiotemporal gait parameters in ambulatory children with CP who are in level I and II of the GMFCS. This study is the first to assess the reliability of the G-Walk sensor system in children with CP.

Test-retest reliability measures the extent of which a testing measure is consistent and repeatable and it involves validation of an assessment over multiple time points. Reliability can be calculated as the ratio of a true score variance to an observed score variance and is often expressed using a correlation coefficient, which ranges from 0 to 1. The closer the coefficient is to 1, the more reliable a testing measure is considered to be, implying that the true score is assessed with little error variance [18].

The results show that the G-Walk had almost perfect reliability in the assessment of spatiotemporal gait parameters and pelvic girdle angles in children with CP. The reliability of the device on spatiotemporal gait parameters and pelvic girdle angles reflects the devices’ ability to provide consistent test-retest measurements.

**Figure 2:**
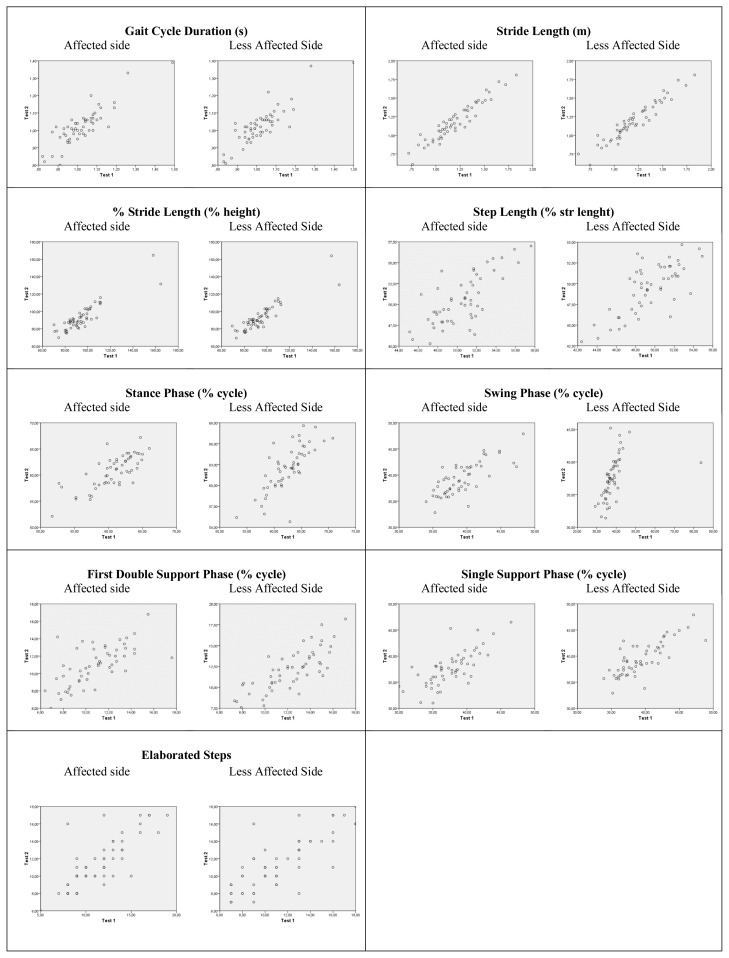
Unity line score plots for gait outcome measures (Test 1-Test 2). Dots on the unity line represent the identical test-retest score. Higher scores on Test 1 appear beneath the line and higher scores on Test 2 appear above the line.

**Figure 3:**
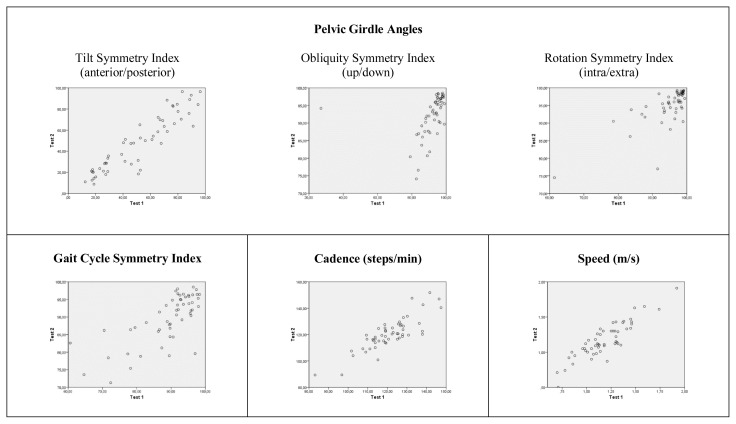
Unity line score plots for basic gait mobility outcome measures (Test 1-Test 2). Dots on the unity line represent identical test-retest score. Higher scores on Test 1 appear beneath the line and higher scores on Test 2 appear above the line.

The reliability levels were calculated using ICCs. In the reliability analysis, the ICC value was above 70%, indicating an acceptable confidence level. The ICC values ranged from 0.799 to 0.977 between consecutive measurements performed in five days in terms in all of the gait parameters. According to the ICC values, the reliability of the G-Walk was confirmed in children with CP. However, when the unity line score plots (Figure 2 and 3) were investigated, it can be seen that almost all plots had approximately 45° slope with the exception of less affected side swing phase and pelvic obliquity symmetry index score. As it can be seen in the respective plots, one subject in each variable has deviated from the mean and we believe the decrease in the slope of these plots is due to the values of the deviated subject.

MDC values can help in identifying a true change in measured performance that is beyond random variations [19]. As a derivative of the intra-class correlation and the standard deviation of the scores, the MDC value provides understanding of the psychometrics of the outcome measure. In this study, the MDC_95_ for the G-Walk parameters show that the G-Walk has little measurement error. High MDC values may raise concerns regarding the accuracy of the outcome measure. For example, high MDC values may show that the outcome measure itself is not specific enough to measure the true capacity, the assessed capacity is not stable from day to day, or that the measured performance is affected by other factors [20,21].

The SEM is a reliability measure that assesses response stability. The SEM is used to estimate the standard error in a set of repeated scores. A gait analysis must be applicable in a clinical setting in order for it to be effective. Thus, it needs to be easy to apply in a variety of life situations and it needs to be reliable [22].

De Ridder et al. have investigated the concurrent validity of the G-walk on gait parameters in healthy subjects. They have concluded that, the G-Walk has excellent concurrent validity for speed, cadence, stride length, and stride duration. Regarding the reliability of the device, the authors have stated that G-Walk is reliable for all measured spatiotemporal parameters. Similar to their findings, we have also found the BTS G-Walk has perfect reliability for measuring these parameters. In addition to the study by De Ridder et al., we have also found that the reliability of the G-Walk on measuring pelvic angles was perfect. In light of the results presented here, a gait analysis carried out with the G-Walk system is a reliable method to assess gait in children with CP in a clinical setting.

Limitations of this study are that the age range of participants included in the study was limited to 5–15 and therefore cannot be generalized to the adult CP population. Also, this study did not include participants who had ataxic, dyskinetic or nonclassifiable type CP. Children who had severe cognitive impairment were not included in the study due to lack of cooperation. This study only aimed to investigate the reliability of the G-Walk. Further investigation regarding the validity of this device must be performed.

In conclusion, the current study established test-retest reliability and MDC values in children with CP who could walk independently. Results show that the G-Walk is reliable for gait assessment in this population.

## Figures and Tables

**Table 1:** Demographic and functional characteristics of children.

	All children (n: 54)		GMFCS Level I	GMFCS Level II
	Mean (SD)	Range		
**Sex (male/female)**			11/17	18/8
**Height (cm)**	133.3 (18.91)	113–168	136.22 (21.93)	135.23 (17.52)
**Weight (kg)**	30.28 (14.28)	13–60	31.06 (14.48)	3..09 (16.36)
**Age (year)**	9.19 (3.49)	5–15	9.56 (4.10)	9.77 (3.22)
**Topographical Classification**	(n)		(n)	(n)
**Unilateral involvement**	32		26	6
**Bilateral involvement**	22		2	20

**SD:** Standard Deviations; **GMFCS:** Gross Motor Function Classification System.

**Table 2:** Means and standard deviations of measurements.

			Test Mean (SD)	Retest Mean (SD)	P
**Cadence (steps/min)**			121.1 (11.9)	120.5 (12.1)	0.501
**Speed (m/s)**			1.19 (0.24)	1.17 (0.25)	0.439
**Gait cycle duration (s)**		Affected side	1.02 (0.11)	1.02 (0.1)	0.813
		Less affected side	1.02 (0.11)	1.03 (0.11)	0.409
**Stride length (m)**		Affected side	1.20 (0.24)	1.19 (0.25)	0.154
		Less affected side	1.20 (0.25)	1.19 (0.25)	0.276
**% Stride length (% height)**		Affected side	94.41 (16.77)	92.75 (15.40)	0.106
		Less affected side	94.32 (16.69)	92.92 (15.23)	0.150
**Step length (% str length)**		Affected side	50.50 (2.86)	50.53 (2.92)	0.912
		Less affected side	49.46 (2.71)	49.47 (2.92)	0.984
**Stance phase (% cycle)**		Affected side	60.72 (3.23)	60.74 (3.09)	0.941
		Less affected side	62.49 (3.32)	62.49 (3.20)	0.986
**Swing phase (% cycle)**		Affected side	39.28 (3.23)	39.25 (3.09)	0.926
		Less affected side	37.49 (3.35)	37.46 (3.22)	0.338
**First double support phase (% cycle)**		Affected side	10.81 (2.40)	10.96 (2.26)	0.607
		Less affected side	12.29 (2.45)	12.09 (2.43)	0.373
**Single support phase (% cycle)**		Affected side	37.61 (3.30)	37.70 (3.33)	0.815
		Less affected side	39.43 (3.32)	39.48 (3.16)	0.848
**Elaborated steps**		Affected side	11.46 (3.06)	11.52 (2.86)	0.832
		Less affected side	11.39 (3.21)	11.57 (3.01)	0.497
**Gait cycle symmetry index**			89.07 (8.70)	89.61 (6.88)	0.509
**Pelvic girdle angles**	Tilt symmetry index (anterior/posterior)		51.80 (25.32)	48.29 (26.29)	0.227
	Obliquity symmetry index (up/down)		92.83 (4.93)	92.26 (5.69)	0.700
	Rotation symmetry index (intra/extra)		94.86 (6.45)	95.06 (4.91)	0.737

**SD:** Standard Deviations, **min:** Minute, **s:** Second, **m:** Meter, **Analysis Duration (s):** Duration of the whole trial, **Cadence (steps/min):** Number of steps in a min, **Speed (m/s):** Average walking speed, **Gait cycle duration (s):** Average value of the time interval between two consecutive heel strikes of the same foot, **Stride length (m):** Average value of distances between each initial contact and the next one of the same side, **% Stride length (%height):** Stride length normalized over the height of the subject, **Step length (% str. length):** Average value of distances between each initial contact and the next one of the contralateral side, **Stance phase (% cycle):** average value of the duration of the right and left foot support phase as percentage of the gait cycle, **Swing phase (% cycle):** Average value of the duration of the right and left swing phase as percentage of the gait cycle, **Double support phase (% cycle):** Average value of the duration of the phase in which both feet are in stance position as percentage of the gait cycle, **Single support phase (% cycle):** Average value of the duration of the phase in which only one foot is in stance position as percentage of the gait cycle, **Elaborated steps:** Number of strides considered in the analysis.

**Table 3:** ICC, SEM, and MDC values of measurements.

Test		ICC^*^	95% CI		SEM	MDC
			Lower bound	Upper bound		
**Cadence (steps/min)**		0.915	0.854	0.951	3.50	9.7
**Speed (m/s)**		0.941	0.898	0.966	0.29	0.8
**Gait cycle duration (s)**	Affected side	0.926	0.872	0.957	0.28	0.78
	Less affected side	0.927	0.875	0.958	0.28	0.78
**Stride length (m)**	Affected side	0.974	0.955	0.985	0.19	0.57
	Less affected side	0.977	0.960	0.986	0.18	3.27
**% Stride length (% height)**	Affected side	0.942	0.900	0.966	3.87	10.73
	Less affected side	0.948	0.910	0.970	3.64	10.09
**Step length (% str length)**	Affected side	0.844	0.730	0.909	1.14	3.16
	Less affected side	0.850	0.741	0.913	1.09	3.02
**Stance phase (% cycle)**	Affected side	0.866	0.769	0.922	1.16	3.22
	Less affected side	0.864	0.765	0.921	1.20	3.33
**Swing phase (% cycle)**	Affected side	0.865	0.766	0.922	1.16	3.22
	Less affected side	0.864	0.764	0.922	1.21	3.35
**First double support phase (% cycle)**	Affected side	0.799	0.653	0.883	1.04	2.88
	Less affected side	0.876	0.788	0.928	0.86	2.38
**Single support phase (% cycle)**	Affected side	0.847	0.736	0.911	1.30	3.6
	Less affected side	0.873	0.781	0.927	1.15	3.19
**Elaborated steps**	Affected side	0.885	0.801	0.933	1.01	2.8
	Less affected side	0.887	0.805	0.934	1.05	2.91
**Gait cycle symmetry index**		0.832	0.710	0.902	3.19	2.84
**Pelvic girdle angles**	Tilt symmetry index (anterior/posterior)	0.954	0.916	0.974	5.53	15.33
	Obliquity symmetry index (up/down)	0.887	0.804	0.934	1.78	4.93
	Rotation symmetry index (intra/extra)	0.830	0.706	0.901	2.35	6.51

**ICC:** Intraclass correlation coefficient, **CI:** Confidence interval, **SEM:** Standard error measurements, MDC: Minimal detectable change, min: Minute, s: Second, m: Meter, **Analysis Duration (s):** Duration of the whole trial, **Cadence (steps/min):** Number of steps in a min, **Speed (m/s):** Average walking speed, **Gait cycle duration (s):** Average value of the time interval between two consecutive heel strikes of the same foot, **Stride length (m):** Average value of distances between each initial contact and the next one of the same side, **% Stride length (%height):** Stride length normalized over the height of the subject, **Step length (% str. length):** Average value of distances between each initial contact and the next one of the contralateral side, **Stance phase (% cycle):** average value of the duration of the right and left foot support phase as percentage of the gait cycle, **Swing phase (% cycle):** Average value of the duration of the right and left swing phase as percentage of the gait cycle, **Double support phase (% cycle):** Average value of the duration of the phase in which both feet are in stance position as percentage of the gait cycle, **Single support phase (% cycle):** Average value of the duration of the phase in which only one foot is in stance position as percentage of the gait cycle, **Elaborated steps:** Number of strides considered in the analysis.
